# Trends in alcohol and marijuana detected in homicide victims in 9 US states: 2004–2016

**DOI:** 10.1186/s40621-019-0229-4

**Published:** 2020-01-06

**Authors:** Oybek Nazarov, Guohua Li

**Affiliations:** 1 Department of Anesthesiology, Columbia University Vagelos College of Physicians and Surgeons, New York, NY USA; 2 Department of Epidemiology, Columbia University Mailman School of Public Health, New York, NY USA

**Keywords:** Homicide, Marijuana, Violence

## Abstract

**Background:**

Use of alcohol and other drugs is a major risk factor for assaultive injuries and violent deaths. The purpose of this study was to examine the time trends in the prevalence of alcohol and marijuana detected in homicide victims.

**Methods:**

We analyzed toxicological testing data for homicide victims (*n* = 12,638) from the 2004–2016 National Violent Death Reporting System in 9 US states (Colorado, Georgia, Massachusetts, New Jersey, Oregon, Rhode Island, South Carolina, Virginia, and Wisconsin). We used the Cochran-Armitage test for trend to assess the statistical significance of changes in the prevalence of alcohol and marijuana detected in these homicide victims during the study period.

**Results:**

Overall, 37.5% of the homicide victims tested positive for alcohol, 31.0% positive for marijuana, and 11.4% positive for both substances. During the study period, the prevalence of marijuana increased from 22.3% (95% confidence interval [CI] = 19.6, 25.0) in 2004 to 42.1% (95% CI = 39.2, 44.9) in 2016 (*Z* = -15.7; *P* < .001) while the prevalence of alcohol declined slightly (*Z* = 1.5; *P* = 0.143). Marked increases in the prevalence of marijuana were observed in both sexes and across age and racial groups.

**Conclusions:**

Marijuana is increasingly detected in homicide victims irrespective of demographic characteristics. Further research is needed to assess the causal role of marijuana use and concurrent use of marijuana and alcohol in homicide victimization.

## Background

Homicide is a major cause of injury mortality, particularly for black adolescents and young adults (Logan et al. 2011). Use of alcohol is an important risk factor for homicide, with nearly half of the victims and 40–50% of the perpetrators testing positive for alcohol (Darke 2010; Kuhns et al. 2011), and is associated with a four-fold increased risk of homicide victimization (Hohl et al. 2017). Goodman et al. (1986) found that in 44% of homicide incidents, both the victims and the perpetrators had consumed alcohol. Another substance commonly involved in homicides is marijuana, which is detected in 6–40% of victims and 5–31% of perpetrators (Darke et al. 2009; Kuhns et al. 2009; Darke 2010; Guimaraes et al. 2017) and is associated with substantially increased risks of homicide victimization (Darke et al. 2009; Temple et al. 2013; Guimaraes et al. 2017).

The mechanisms linking alcohol use to increased homicide risk have been studied extensively. For instance, the pharmacological disinhibition model posits that alcohol intoxication impacts the area of the brain responsible for impulse control, judgment and interpretation of social cues, resulting in impulsive or aggressive behaviors (Exum 2006). Similarly, it is evident that marijuana use impairs cognitive functions, inhibits impulse control, and increases aggressive behaviors (Yanowitch and Coccaro 2011; Temple et al. 2013). Moreover, withdrawal from chronic marijuana use may instigate irritability and heighten the risk of conflict and aggression (Smith et al. 2013).

Despite the growing body of evidence linking alcohol and marijuana to homicide victimization, there is a dearth of information about the contemporary trends in the prevalence of alcohol and marijuana among homicide victims in the United States (Kuhns and Maguire 2012; Delaveris et al. 2014). As of June 2019, 34 states and the District of Columba have legalized marijuana for medical use; of them, 11 states and the District of Columbia have also legalized recreational marijuana for adults aged 21 years and older (NCSL 2019). Given the increased permissibility and availability of marijuana, it is necessary to closely monitor its potential adverse health consequences, particularly its involvement in injuries. This study assessed time trends in the prevalence of alcohol and marijuana detected in homicide victims in 9 US states from 2004 through 2016.

## Methods

Data for this study came from the 2004–2016 National Violent Death Reporting System (NVDRS). The NVDRS is a population-based surveillance system for violent deaths occurring in the United States and is administered by the National Center for Injury Prevention and Control, Centers for Disease Control and Prevention (CDC 2015). Violent deaths refer to fatalities resulting from the intentional use of physical force, power, or other means against others or oneself. Incepted in six states in 2002 to better understand and prevent homicide and suicide, the NVDRS culls data from death certificates, coroner/medical examiner records including toxicology reports, and law enforcement reports. In 2018, the NVDRS was expanded to all 50 states, the District of Columbia, and Puerto Rico, serving as a source of data for violence research and prevention (CDC 2018; Nazarov et al. 2019).

### Study sample

The study sample included homicide victims in 9 states that have been participating in the NVDRS since 2004 (Colorado, Georgia, Massachusetts, New Jersey, Oregon, Rhode Island, South Carolina, Virginia, and Wisconsin). Alabama, Maryland, North Carolina, and Oklahoma, which have been participating in the NVDRS since 2004, were excluded from this study because toxicological testing for marijuana in these states was performed on less than 5% of homicide victims. During the study period, the NVDRS recorded a total of 30,433 homicide victims in the 9 states. Excluded from the analysis were 1518 victims under 15 years of age due to sparse data and 16,277 victims with missing or incomplete toxicological testing data. Available for analysis were 12,638 homicide victims aged 15 years and older with complete toxicological testing data.

### Measures

Drug tests were performed on blood, urine, vitreous humor (ocular fluid), and/or bile by using head-space gas chromatography, ELISA, and mass spectrometry (CDC 2015). We categorized and coded alcohol and marijuana according to the NVDRS Web Coding Manual (CDC 2015). Blood alcohol concentrations (BACs) were measured in grams per deciliter, and BACs of 0.01 g per deciliter or greater were considered alcohol-positive.

### Statistical analysis

The prevalence of alcohol and marijuana detected in homicide victims was calculated by the calendar year and victim characteristics. The Cochran-Armitage test for trend was used to assess the statistical significance of trends in binomial proportions of substance positivity analyzed in this study. Statistical significance was set at *P* < .05 for 2-tailed tests. Data analysis was performed using SAS, version 9.4, software (SAS Institute, Inc., Cary, North Carolina).

## Results

Homicide victims tested for alcohol and drugs were more likely than those not tested to be white (38.3% vs 31.7%; *P <* .001). The two groups did not differ significantly in age and sex distributions and incident circumstances such as a fight between two people, drug dealing or death due to the firearm that subsequently led to death.

Of the 12,638 homicide victims with toxicological testing results, 37.5% [95% confidence interval (CI) = 36.7, 38.3] were positive for alcohol, 31.0% (95% CI: 30.2, 31.8) were positive for marijuana, and 11.4% were positive for both substances (95% CI = 10.8, 11.9). Among those testing positive for alcohol, the mean BAC was 0.13 g/dL (standard deviation = 0.10 g/dL), and 63.9% were legally impaired (i.e., BAC ≥ 0.08 g/dL).

The prevalence of alcohol and marijuana varied significantly with demographic characteristics (Table 1). Male victims were more likely than female victims to test positive for alcohol, marijuana, and both substances. Substantially elevated prevalence of alcohol was found in victims aged 35–49 years and in white victims. The prevalence of marijuana was highest in victims aged 15–20 years (46.8%) and decreased progressively with age. Black victims had a considerably higher prevalence of marijuana (38.0%) than white victims (23.2%) and other victims (23.4%) (Table 1).

**Table 1:** Prevalence of alcohol and marijuana detected in homicide victims by demographic characteristics: National Violent Death Reporting System; Colorado, Georgia, Massachusetts, New Jersey, Oregon, Rhode Island, South Carolina, Virginia, and Wisconsin; 2004–2016

Characteristics	Homicide victims	Positive for alcohol		Positive for marijuana		Positive for both substances	
	No.	No.	%	No.	%	No.	%
Age, years *							
15–20	1946	450	23.1	910	46.8	223	11.5
21–34	5839	2342	40.1	2253	38.6	852	14.6
35–49	2872	1287	44.8	564	19.6	280	9.8
50–64	1418	543	38.3	177	12.5	76	5.4
≥ 65	555	111	20.0	14	2.5	5	0.9
Sex *							
Male	10,303	4094	39.7	3567	34.6	1322	12.8
Female	2335	643	27.5	351	15.0	114	4.9
Race *							
White	4691	1874	40.0	1090	23.2	470	10.0
Black	6659	2412	36.2	2529	38.0	860	12.9
Other	972	348	35.8	227	23.4	86	8.9

*Significant at *P* < .001 level for alcohol, marijuana and both substances

**Fig. 1:**
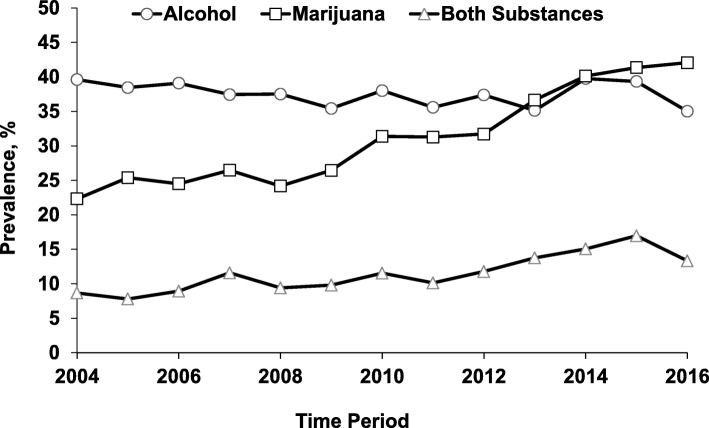
Prevalence of Alcohol and Marijuana Detected in Homicide Victims by Calendar Year: National Violent Death Reporting System; Colorado, Georgia, Massachusetts, New Jersey, Oregon, Rhode Island, South Carolina, Virginia, and Wisconsin; 2004–2016. *Note.* Cochran-Armitage χ^2^ test for trend: alcohol (*Z* = 1.46; *P* = .143); marijuana (*Z* = -15.67; *P* < .001); both substances (*Z* = -7.90; *P* < .001)

**Fig. 2:**
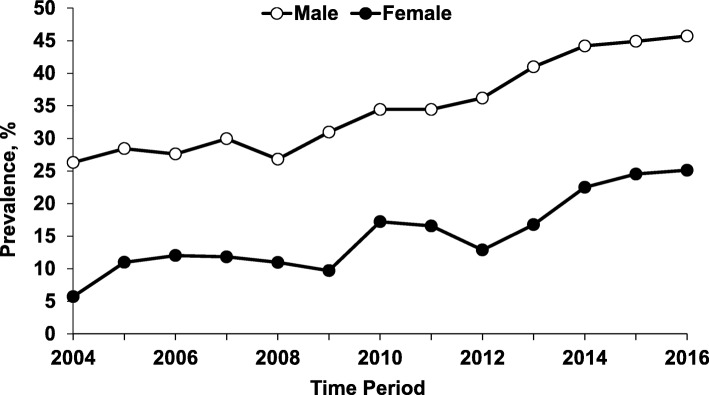
Prevalence of Marijuana Detected in Homicide Victims by Year and Sex: National Violent Death Reporting System; Colorado, Georgia, Massachusetts, New Jersey, Oregon, Rhode Island, South Carolina, Virginia, and Wisconsin; 2004–2016. *Note.* Cochran-Armitage χ^2^ test for trend: male (*Z* = -14.17; *P* < .001); female (*Z* = -7.01; *P* < .001)

**Fig. 3:**
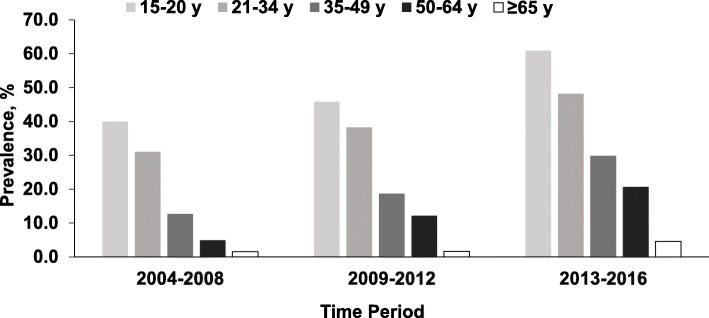
Prevalence of Marijuana Detected in Homicide Victims by Year and Age: National Violent Death Reporting System; Colorado, Georgia, Massachusetts, New Jersey, Oregon, Rhode Island, South Carolina, Virginia, and Wisconsin; 2004–2016. *Note.* Cochran-Armitage χ^2^ test for trend: 15–20 years (*Z* = -7.10; *P* < .001); 21–34 years (*Z* = -11.15; *P* < .001); 35–49 years (*Z* = -9.51; *P* < .001); 50–64 years (*Z* = -7.41; *P* < .001); ≥ 65 years (*Z* = -1.84; *P* = .065)

**Fig. 4:**
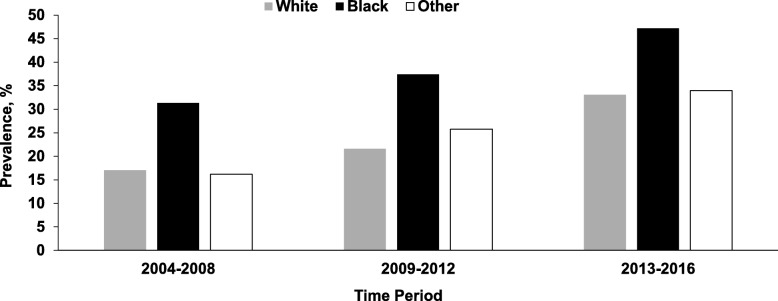
Prevalence of Marijuana Detected in Homicide Victims by Year and Race: National Violent Death Reporting System; Colorado, Georgia, Massachusetts, New Jersey, Oregon, Rhode Island, South Carolina, Virginia, and Wisconsin; 2004–2016. *Note.* Cochran-Armitage χ^2^ test for trend: white (*Z* = -10.34; *P* < .001); black (*Z* = -10.93; *P* < .001); other (*Z* = -5.04; *P* < .001)

During the study period, the prevalence of marijuana detected in homicide victims almost doubled, increasing from 22.3% (95% CI = 19.6, 25.0) in 2004 to 42.1% (95% CI = 39.2, 44.9) in 2016 (*Z* = − 15.7, *P* < 0.001), while the prevalence of alcohol declined slightly from 39.6% in 2004 to 35.0% in 2016 (*Z* = 1.46; *P* = 0.143) (Fig. 1). Due to the divergent trends, the prevalence of marijuana surpassed the prevalence of alcohol in 2013 (Fig. 1). The prevalence of marijuana increased in both sexes but the increase was more pronounced in female victims (from 5.7% in 2004 to 25.1% in 2016) (*Z* = − 7.01, *P* < 0.001) (Fig. 2). The prevalence of marijuana detected in homicide victims increased across age and racial groups (Figs. 3 and 4).

## Discussion

Our results indicate that the prevalence of marijuana among homicide victims almost doubled during the 2004 through 2016 study period while the prevalence of alcohol declined slightly. As a result, marijuana has become the most commonly detected substance in homicide victims. In 2016, over 42% of homicide victims tested positive for marijuana and the prevalence was especially high in adolescent victims (68.5%) and in black victims (47.9%). During the study period, the prevalence of alcohol remained fairly stable at about 38% and the prevalence of concurrent use of marijuana and alcohol increased by 53.9%. Although demographic distributions of alcohol detected in homicide victims are consistent with drinking behavior in the general population, the epidemiologic patterns of marijuana detected in homicide victims differ markedly from the results of the National Survey on Drug Use and Health (Azofeifa et al. 2016). Specifically, the prevalence of marijuana detected in homicide victims is about three times the prevalence of self-reported past-year marijuana use in the general population and has increased more rapidly than in the general population. Moreover, the age and race gradients in the prevalence of marijuana detected in homicide victims, particularly the excess prevalence detected in adolescent victims and black victims, are much more pronounced than in the general population. These findings are of potential public health significance because previous research has established alcohol use and marijuana use as risk factors for violent death (Wolfgang 1957; Goldstein 1986; Sampson and Lauristen 1994; Howell 1998; Galea et al. 2002; Darke and Duflou 2008; Auckloo and Davies 2019). If confirmed, results of our study indicate that marijuana is playing an increasingly important role in homicide victimization.

The marked increase in the prevalence of marijuana reported in the present study is germane to the growing decriminalization of marijuana (NCSL 2019). Although medical marijuana laws do not seem to have a measurable impact on adolescent marijuana use, legalizing recreational marijuana is associated with the increased use of marijuana among youth (Rusby et al. 2018; Koval et al. 2019). Given that more states are considering to legalize recreational marijuana, it is necessary to develop surveillance systems for monitoring the exposure and health consequences related to marijuana.

### Limitations

This study has several notable limitations. First, toxicological testing and reporting varied by states. The cross-state variations become particularly problematic for those states with independent county coroner systems rather than a centralized medical examiner system. Second, in order to enhance internal validity, we restricted the study to 9 states that have been participating in the NVDRS since 2004 and that have performed toxicological testing on at least 20% of homicide victims. Given that the 9 states studied account for only about 17.2% of the US total population and toxicological testing data were unavailable for the majority (56.3%) of homicide victims, our results may not be generalizable to the nation and be susceptible to selection bias. Third, the NVDRS does not record the delta-9-tetrahydrocannabinol level and information about the timing and dosage of marijuana use. The detection window for marijuana is up to 3 weeks in blood and up to 1 month in the urine (Hound Labs 2019). Hence, positive results of toxicological testing indicate marijuana use but do not necessarily imply marijuana-induced impairment. Fourth, the overall increase in marijuana positivity might be partially attributed to improved toxicological testing procedures and increased marijuana use in the general population as marijuana becomes more permissible and more accessible. Fifth, data on the type of marijuana use (medical or recreational) were not available. Thus, the marked increase in marijuana in homicide victims reported in this study cannot be attributed to any particular source of marijuana procurement. Finally, we restricted our analysis to the two most commonly used substances – alcohol and marijuana. Other drugs, such as cocaine, amphetamines, and opiates, may also contribute to homicide victimization although they are less frequently detected in homicide victims than alcohol and marijuana. Despite these limitations, this study provides valuable evidence about the upward trends in recent years in the prevalence of marijuana use and concurrent use of alcohol and marijuana detected in homicide victims. A more comprehensive analysis based on the entire NVDRS data system is warranted to corroborate the findings of this study and shed light on other drugs, such as opiates, methamphetamines, and cocaine.

## Conclusions

There has been a substantial increase in the prevalence of marijuana use detected in homicide victims across demographic groups in the United States between 2004 and 2016. Since 2013, marijuana has surpassed alcohol to become the most frequently detected substance in homicide victims. During the study period, the prevalence of concurrent use of alcohol and marijuana detected in homicide victims has also increased significantly. Our findings indicate that marijuana use is increasingly detected in homicide victims and that the role of marijuana plays in homicide victimization needs to be rigorously examined.

## Data Availability

The National Violent Death Reporting System data are available from the Centers for Disease Control and Prevention, National Center for Injury Prevention and Control, Division of Violence Prevention.

## Abbreviations

BAC: Blood alcohol concentration
CI: Confidence interval
NVDRS: National Violent Death Reporting System
